# Naughty Children

**Published:** 1902-05-03

**Authors:** 


### The Hospital

A Journal of

The Medical Sciences and Hospital Administration.

Vol. XXXII.—No. 814.] Edited by Sir Henry Burdett, K.C.B., and Solomon C. Smith, M.D., M.R.C.P. [May 3, 1902.

### Naughty Children.

It would be difficult to overestimate the importance of the subject dealt with by Dr. Still in the Gulstonian Lectures recently delivered before the Royal College of Physicians, under the somewhat wide and indefinite title of "Some Abnormal Psychical Conditions in Children." It has long been recognised that defective moral control is apt to occur in association with those disorders of intellect which are ordinarily recognised as idiocy, imbecility, or insanity, and no one doubts the morbid nature of the moral defect in these cases. Whether it be regarded as dependent upon the intellectual failure or not, it is clearly part and parcel of the malady, and according to our conception of the processes going on in disorder of mind, so will be our conception of the associated disorder of the moral sense. If the one be regarded as due to disease or imperfection of brain tissue, so also will the other. But children are occasionally met with who exhibit defects of moral control precisely analogous to those which occur with admittedly morbid brains, yet who, so far as ordinary tests go, pass for children of normal intellect; and the question is whether these naughty children are not naughty because of defect in the physical substratum of morality, if we may use such a phrase, just as imbeciles are defective in the physical substratum of intellect. For a definition of the term moral control, Dr. Still adopts the statement that it is "the control of action in conformity with the idea of the good of all," which at any rate is sufficiently comprehensive, and he specially warns us that he by no means limits the terms "moral" or "immoral" to sexual relations.

Although defect of moral control is very often seen in connection with idiocy or imbecility, it is to be noted that there is little relation to be discovered between the degree of the defect on the intellectual and the moral sides. The drivelling idiot who recognises no one, does not distinguish his food, and is a mere automaton, is, of course, devoid of the higher attribute which we term moral control. But when we come to the higher grades of imbeciles, in which some comparison of the various faculties is possible, it is obvious that just as the defect in the more intellectual faculties is by no means evenly distributed, so that, for example, a child who may be able to perform wonderful arithmetical tricks may be almost an idiot in other directions, so there may be, and indeed is, no relation whatever between the degrees of defect exhibited in the intellectual faculties and in the moral control. In Dr. Still's words, "whilst defect of moral control is often associated with general impairment of intellect, there is no constant proportion between them." Thus, although it is probably true that cell-modification dependent upon interference with cell-nutrition is the physical basis of moral defect, this modification must occur in the finest processes and be something quite different from such gross lesions as, for example, are found in certain cases of idiocy. Dr. Still then goes on to consider the cases in which morbid defect of moral control occurs in relation with physical disorder, and shows that such defect may arise not only from arrest or delay in its development by physical disease occurring in infancy, but that, even after considerable progress has already been made in its development, it may be lost to a greater or less degree as the result of physical maladies, particularly lesions of the brain and certain febrile conditions, and this even in cases in which the intellect appears intact.

Can we not then go further and place certain incorrigibly naughty children in the same category, as the victim of morbid brain tissue, even though they show no sign at all of defective intellect? There are children who lie and steal without reason, are cruel to animals, are dangerous to leave with other children lest they should injure them, and who commit the same misdemeanour time after time within a few hours after punishment, notwithstanding that they may have been greatly affected by the punishment at the time; yet these children may show no sign of intellectual deficiency. Surely the defect of moral control in such cases, whatever be its cause, is of the same nature as that so frequently seen in cases of obvious intellectual deficiency. But Dr. Still goes further and shows that defect of moral control, while sometimes permanent, may be only temporary, in some cases passing away after an outburst never to return, while in others periods of defective moral control may alternate with periods in which no such defect is present. Here we seem to come to the brink of a moral insanity, and it would be easy to follow the lead given by Dr. Still, and to discuss the question of the relation not only of some of the acute forms of insanity but of these cases of defective moral control in children, with the presence of toxins in the blood and their injurious action on those finer nerve couplings which are brought into operation in all mental action, including the moralities. The matter is one of much practical interest. In regard to the more temporary attacks of moral defect, modern pathology by its teaching in regard to toxins would seem to give much support to the method of the old schoolmaster who said that when he found a boy incorrigibly naughty he had recourse to Gregory powder; while in regard to the general scheme of education to be adopted in the case of naughty children one cannot but feel, in view of the marvellous improvement which is produced in the intellectual faculties by early and judicious teaching, that perhaps an equally careful training of that residuum of moral control which is still to be found in all, might rescue some of those passionate, spiteful, lawless, shameless children, whose condition is allied to moral imbecility, from the sad future that is before them.

